# Effectiveness of Mindfulness-Based Stress Reduction in a Self-Selecting and Self-Paying Community Setting

**DOI:** 10.1007/s12671-017-0873-0

**Published:** 2017-12-19

**Authors:** Lise Juul, Karen Johanne Pallesen, Jacob Piet, Christine Parsons, Lone Overby Fjorback

**Affiliations:** 1 Department of Clinical Medicine, Danish Center for Mindfulness, Aarhus University, Jens Chr. Skous Vej 4, 8000 Aarhus, Denmark; 2 School of Culture and Society, Interacting Minds Centre, Aarhus University, Aarhus, Denmark

**Keywords:** Mindfulness, Mindfulness-Based Stress Reduction, MBSR, Community setting, Effectiveness, Implementation

## Abstract

We aimed to evaluate the effectiveness of Mindfulness-Based Stress Reduction (MBSR) when implemented in a community setting as a self-referred and self-paid course. Pre-post changes and Cohen’s *d* effect sizes were calculated for questionnaire measures of mindfulness, perceived stress, and symptoms of anxiety and depression. We compared these effect sizes with those from intervention groups in randomized controlled trials (RCTs), with populations similar to our study sample. These RCTs reported significant effects of MBSR compared to control condition. MBSR was delivered in three different Danish cities by ten different MBSR teachers with various professional backgrounds and MBSR teaching experience. One hundred and thirty-two participants were included in the study: 79% were women, mean age 45 ± 10.4 years, 75% of the participants had more than 15 years of education, 38% had a Perceived Stress Scale (PSS) score≥18, and 27% had a history of mental disorder. Post MBSR, the proportion of participants with a PSS≥18 decreased by 16% points (95%CI −26 to −6), *p* = 0.0032. Within-group effect sizes for (i) the total study population (ii) the subgroup with PSS≥18 at baseline (iii) intervention group in reference RCTs were as follows: PSS: *d* = 0.50:1.47:1.00, Symptom Check List 5: *d* = 0.48:0.81:0.77, Five Facet Mindfulness Questionnaire: *d* = 0.67:1.09:1.00. Our results showed that MBSR was effective. The effects were largest among the participants reporting highest perceived stress level at baseline. Our participants were mainly women who were middle-aged, with high educational levels, and more perceived stress and a greater history of mental disorder than the general population, and who were able to seek out and pay for an MBSR course. Reaching vulnerable groups with a clear need for stress management will, however, require other implementation strategies.

## Introduction

Long-term stress is an increasing public health problem and is associated with impaired psychological and physical functioning (Chandola et al. 2006; Horri et al. 2010; Kelly and Ismail 2015; Pallesen et al. 2016; Rosengren et al. 2004; Stansfeld et al. 2002). With long-term stress, there is a risk of poor health behavior, which further contributes to reduced health (Ng and Jeffery 2003; Rod et al. 2009; Stults-Kolehmainen and Sinha 2014). Perceived stress is associated with mortality. For example, a recent Danish population-based study showed that higher perceived stress was associated with higher mortality within a 4-year follow-up, compared to lower perceived stress (Prior et al. 2016).

Stress is an adaptive psychophysiological response to experienced challenges (Mc Ewen and Norton Lasley 2002). In acute situations, stress is beneficial as it helps the body to establish energy resources. During long-term on-going stress, in contrast, the stress response causes wear and tear on the body, as restitution is put on hold (Mc Ewen and Norton Lasley 2002; Pallesen et al. 2016). Another consequence of long-term stress can be physiological changes that have wide-ranging harmful effects on bodily functions. One example, only recently identified, is decreased physiological sensitivity to cortisol, which occurs as this stress hormone is present in the blood stream for extended periods of time (Cohen et al. 2012). In this process, a gradual decrease in cortisol receptors on cell surfaces occurs throughout the body, and the signaling value of cortisol is consequently diminished. A central effect of this “cortisol resistance” is the triggering of inflammation (normally downregulated by cortisol), which is a critical early step in a multitude of symptoms and diseases (Cohen et al. 2012). This change in the signaling power of cortisol also affects glucose metabolism and may lead to overweight and diabetes (Joseph and Golden 2016).

The Mindfulness-Based Stress Reduction (MBSR) program was developed by Jon Kabat-Zinn in 1979 at the University of Massachusetts Medical School. The program components and structure are well described (Kabat-Zinn 1990; Kabat-Zinn et al. 2017; Santorelli 1999, 2014). A recent systematic review of previous reviews (*N* = 23) examined the accumulating evidence for the effects of MBSR (Gotink et al. 2015). This review, including 115 randomized controlled trials (RCTs) suggested that MBSR had beneficial effects on stress, anxiety, depression, quality of life and physical functioning and could be an effective adjunct for conditions such as cancer, cardiovascular disease, chronic pain, depression, and anxiety. Furthermore, results supported the use of MBSR in symptom prevention in healthy adults and children. This review suggested that MBSR delivered as intended is beneficial for broad range of people. RCTs of MBSR have further shown effects in terms of functional alterations in brain regions involved in how we respond to negative and/or stressful events (Davidson et al. 2003; Farb et al. 2013), changes in associated somatic measures including immune function (Davidson et al. 2003; Rosenkranz et al. 2013) and blood pressure (Hughes et al. 2013; Nyklicek et al. 2013).

While these trials suggest MBSR efficacy, a remaining question concerns the extent to which these results can be achieved in community settings. For MBSR to have a place in public health provision, the program must be implemented with fidelity and accepted by the general target population with stress-related problems, independent of factors such as gender and educational level (Onken et al. 2014). Onken et al. have proposed that the intervention development process is incomplete until an intervention is effective under “usual circumstances” and reaches the intended target population. In order to link basic and applied research, they developed the National Institutes of Health (NIH) Stage Model; an iterative, recursive model of behavioral intervention development that includes six stages with different types of research endeavor (Onken et al. 2014). Stage 0 encompasses basic research about the underlying problems and mechanisms and provides the basis for the development of new interventions or modification of existing ones. Stage 1 research includes identifying intervention components, developing and pilot testing complex interventions. Stage 2 research consists of testing interventions in research settings with research providers: traditional efficacy research. Stage 3 research consists of efficacy testing of interventions in community settings. Stage 4 research consists of testing interventions under normal circumstances delivered by community providers in community settings: effectiveness research. Stage 5 is implementation and dissemination research and focuses on the incorporation of scientifically supported interventions into community settings. Stages 1–5 also encompass basic science on mechanisms of change or the validation of mechanisms of change at the different stages (Onken et al. 2014).

Most studies on the effect of MBSR have been carried out as pilot studies or efficacy trials in research settings, for example with the researcher as the only intervention provider (stages 1 and 2 research) (Dimidjian and Segal 2015). Dimidjian and Segal have pointed out the lack of effectiveness and dissemination studies of MBSR (less than 1% of MBSR research studies at stages 4–5) and highlight the importance of this type of research in order to investigate and improve the public health impact of the program. There is general consensus that the RCT is the gold-standard design for efficacy testing (Bonell et al. 2012; Zwarenstein et al. 2008). A pragmatic randomized trial is also an appropriate solution for testing effectiveness (Zwarenstein et al. 2008). However, randomization is not always possible. Moreover, trial activities such as randomization will always influence participants’ behavior and thereby the effectiveness results to some extent. On the other hand, a lack of control group and solely comparing only pre-post measures means changes could be caused by factors other than the intervention, including “regression towards the mean.” Hence, other valid alternative designs are needed.

In the field of diabetes prevention research, an effectiveness study named “The GOAL Implementation trial” investigated whether results from RCTs that had shown efficacy, for example results from the randomized “Diabetes Prevention Study” (DPS) could be replicated in routine health care (Absetz et al. 2007). The same outcome measures were used at 1-year follow-up, and the study population in “the GOAL study” was compared with the intervention group in the DPS efficacy trial. The randomized DPS efficacy trial had shown statistically significant differences in these outcomes between the intervention and the control group (Tuomilehto et al. 2001). Hence, the underlying rationale of this research design was that benefits/changes/effect sizes achieved in a study population that is comparable with the benefits/changes/effect sizes achieved in an intervention group that have differed statistically significantly from a control group in a RCT (reference trial) were valid findings of effectiveness. In most countries today, there is no access to MBSR in the health care system. High-quality effectiveness studies (stage 4) are important for the decision-making process prior to broad dissemination. Thus, the aim of this study was to evaluate the effectiveness of MBSR when implemented as a self-referred and self-paid course in community settings by a group of MBSR teachers in a design inspired by “the GOAL Implementation Trial.” We conceptualize this evaluation as targeting stage 4 of the NIH Stage Model. We also describe key characteristics of the participants self-selecting to participate in MBSR in Denmark.

## Method

### Participants

**Fig. 1:**
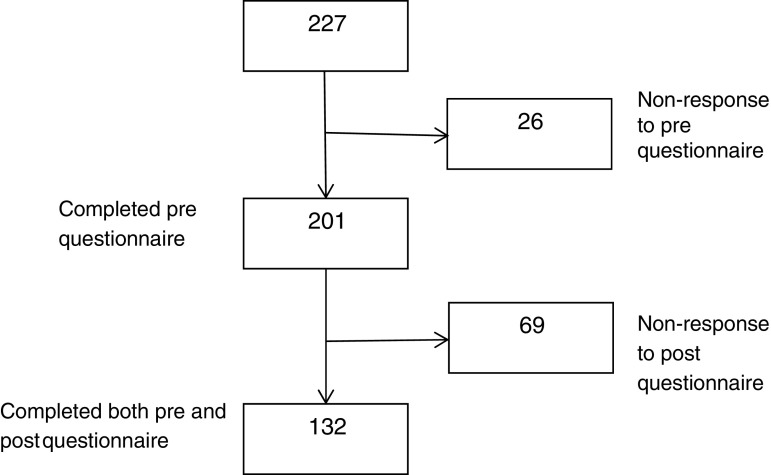
Flowchart

We included 132 participants from an original sample of 227 from the community that self-selected to participate in MBSR offered by Danish Centre for Mindfulness, Aarhus University in the calendar year 2016. Participants completed both the pre- and the post online questionnaires (see Fig. 1).

### Procedure

#### The Context and the Intervention

Danish Centre for Mindfulness was established at Aarhus University in 2013 to provide MBSR teacher education in accordance with international standards outlined by Center for Mindfulness, University of Massachusetts Medical School. Currently, the Danish Centre for Mindfulness offers MBSR to participants in the community for a participation fee of 467 €. MBSR is not currently part of the Danish health care system, which is publicly funded by taxes and ensures all citizens free health care. The structure, methods, and key characteristics of the MBSR program are well described (Kabat-Zinn 1990; Santorelli 2014). The group format, time frame, teaching components, use of exercises, dialog, and inquiry are curriculum based, but also flexible as in other complex interventions. The principles and standards for the MBSR program (Santorelli 2014) and the MBSR teacher training (Kabat-Zinn et al. 2017) are designed to ensure intervention integrity and appropriate delivery of key components.

The MBSR program was delivered as recommended in 2.5-h weekly group sessions over 8 weeks with one 7-h silence retreat day and 45–60 min of daily homework 6 days a week (Kabat-Zinn 1990; Santorelli 1999, 2014). It was delivered by MBSR teachers trained according to international standards (Kabat-Zinn et al. 2017). Ten teachers (LOF, JP are co-authors of this paper) independently delivered the MBSR program in three different cities in Denmark, Aarhus, Gentofte and Holbaek, in groups ranging from 9 to 27 participants. The background professions of the teachers were: psychiatrist (*n* = 1), psychologist (*n* = 6), occupational therapist (*n* = 1), anthropologist (*n* = 1), and other (*n* = 1); and their experience of teaching MBSR ranged from 1 to 10 years, with a median of 2 years.

#### Research Design

We used a quasi-experimental study design estimating pre-post changes and standardized effect sizes, which were compared with effect sizes in intervention groups from relevant reference RCTs. We identified three RCTs as relevant reference trials. In the systematic review of reviews (Gotink et al. 2015), we identified one systematic review (de Vibe et al. 2012) to be the most relevant review for comparison with our study, as it included mixed study populations (healthy adults, adults with chronic diseases, people with perceived stress). We aimed to compare our study population with an intervention group from an efficacy trial including participants with perceived stress, with no specific diagnoses or professional group (e.g., teachers, nurses). In the review by de Vibe, we identified two relevant reference RCTs (de Vibe and Moum 2006; Nyklicek and Kuijpers 2008), which included participants with perceived stress, and which had used the Symptom Check List (SCL)-5 (Tambs and Moum 1993) and the Perceived Stress Scale (PSS) (Cohen et al. 1983), as outcome measures. The other studies in the review had either study populations with specific characteristics such as diagnoses or professions or had used other outcome measures. In a review (Khoury et al. 2015) published after the review of reviews (Gotink et al. 2015), we identified a relevant reference RCT (Robins et al. 2012) that also included participants with perceived stress and had used Five Facet Mindfulness Questionnaire (FFMQ) (Gu et al. 2016) as an outcome measure. The other included studies in that review also had either study populations with specific characteristics or had used other outcome measures. All three reference RCTs had shown a positive effect of MBSR compared to a wait-list control group. The reference RCTs differed with regard to payment for the courses. In the trials by Robins et al. and by Nyklicek et al., MBSR was offered for free, whereas MBSR was self-paid in the trial by de Vibe et al.

### Measures

Data were collected online with an electronic compilation of questionnaires. A link to the questionnaire was sent to the participants 1 week before the start of the program and 1 week after the end of the program. The questionnaire encompassed questions about gender, age, cohabitation, education, employment, chronic diseases, mindfulness, perceived stress, symptoms of anxiety, and depression. Education was categorized in three groups: (1) mandatory school (1 year of preschool class and up to and including 9. grade) and an optional 11th year of school at the most (≤ 11 years), (2) secondary education and/or vocational training < 3 years at the most (>11 < 16 years) and, (3) secondary education and vocational training ≥ 3 years or tertiary education (≥ 16 years).

#### The Five Facet Mindfulness Questionnaire

The Five Facet Mindfulness Questionnaire (FFMQ-15) was used to measure participants’ mindfulness (Gu et al. 2016). The five facets were observing, describing, acting with awareness, non-judging of inner experience, and non-reactivity to inner experience, consisting of three items each. The items had five response categories ranging from “1 = never or very seldom true” to “5 = very often or always true.” The sub- and the total scores were estimated according to instruction. Higher scores indicate higher levels of mindfulness. The internal consistency and the sensitivity to change have previously been found to be adequate for the FFMQ-15 (Gu et al. 2016). The Cronbach’s α was 0.88 in the present study sample.

#### The Perceived Stress Scale

The Perceived Stress Scale (PSS-10) was used to measure the degree to which an individual has perceived life as unpredictable, uncontrollable, and overloading during the previous month (Cohen et al. 1983). The PSS has been widely used in studies of stress reduction in MBSR (de Vibe et al. 2012; Gotink et al. 2015). The 10 items had five response categories ranging from “0 = never” to “4 = very often.” The scores were estimated according to procedure in a total score in the range of 0–40 with higher scores indicating higher perceived stress. We pre-defined a cut-point of ≥18 because a recent Danish population-based study has shown that this cut-point is associated with an increased mortality risk compared to a perceived stress level of below 7 (Prior et al. 2016). At baseline, 50 participants in this study population had a PSS ≥ 18, a total of 80 had PSS < 18, and 2 had missing values. The Cronbach’s α was 0.89 in the present study sample.

#### The Symptom Check List-5

The Symptom Check List-5 (SCL-5)**,** a short form of the Hopkins Symptom Check List-90, was used to measure symptoms of anxiety and depression (Tambs and Moum 1993). This measure has previously been used as a measure of MBSR effects (de Vibe and Moum 2006). In our questionnaire format, the items of the SCL-5 had five response categories; “1 = not at all,” “2 = a little bit,” “3 = some,” “4 = quite a bit,” and “5 = extremely.” However, the original scoring of the SCL-5 measure includes only four response categories; “1 = not at all,” “2 = a little bit,” “3 = quite a bit,” and “4 = extremely.” We made the assumption that half of the responders in our study that had chosen the category “3 = some” would have chosen the category “2 = a little bit,” and the other half would have chosen the category “3 = quite a bit,” if we had used the original version with four response categories. Therefore, we recoded our category “3 = some” to be 2.5. The “quite a bit” and the “extremely” response categories were recoded to be 3 and 4, respectively. We found this the best solution in order to compare our results with prior studies. The score was calculated as the average of the five items. A SCL-5 score >2 has been found to predict the presence of a mental illness, as assessed independently by psychiatrists (Strand et al. 2003). The Cronbach’s α was 0.88 in this study sample. All the questionnaires had been translated into Danish previously according to the WHO-guideline including forward—and expert panel back-translation (WHO 2017).

### Data Analyses

Differences in the pre and post scores of FFMQ, PSS, and SCL-5 were calculated with 95% confidence intervals and *p* values using paired t-tests, both among the total study population and among the pre-defined subgroup with PSS ≥ 18 at baseline. An alpha value of 0.05 was used. Furthermore, Cohen’s *d* were calculated according to the formula: (M post – M pre) / ((sd post + sd pre)/2) (Lakens 2013), for our study groups and for the intervention groups in the three reference RCTs. Questionnaire non-responder analyses were performed for age, gender, educational level, cohabitation, employment, history of mental disorder, and pre-scores of FFMQ, PSS, and SCL-5 by t-test and chi_2_-test. In order to assess the potential impact of non-responding on the estimates of MBSR effect, we performed an analysis assuming that non-responders had no change from baseline post MBSR. We used their pre-MBSR scores as post measures. As two of the ten MBSR teachers were co-authors, and also had the greatest amount of teaching experience, we performed a subgroup analysis, comparing the pre-post changes for participants from MBSR courses delivered by these two teachers with the pre-post changes for participants from MBSR courses delivered by the other eight MBSR teachers. Furthermore, subgroup analyses were performed comparing the pre-post changes in (i) gender, (ii) in participants with self-reported current or previous mental disorder vs. no self-reported mental disorder, and (iii) in participants with tracked attendance data vs. no tracked attendance data.

## Results

### Characteristics of the Included Population and Effectiveness

Figure 1 shows the flowchart of participant inclusion. A total of 132 participants completed both the pre- and the post course questionnaires. Characteristics of the study population are shown in Table 1. The majority of participants were women (79%), and the mean age was 45 ± 10.4 years. A total of 75% of the participants had more than 15 years of education. Among the Danish adult population, 31% have high educational level (Larsen et al. 2014). The majority lived with a partner and/or their children. Only a few were unemployed (7%), retired or on disability pension (3%). At the program start, 14% were on sick leave. A total of 27% reported having or having had a mental disorder. For context, in a national health survey among the Danish adult population, the proportion with self-reported present or previous mental disorder was 14% (95%CI 13.4 to 14.3). Among the women, it was 17% (95% CI 16.4 to 17.8), and among women aged 35–54 years, it was 19% (95% CI 18.0 to 20.5) (Larsen et al. 2014). Twenty-nine percent had, or had previously had, migraine, 13% had osteoarthritis, and 16% had or had previously had a prolapse (Table 1). Before MBSR, the mean PSS score was 15.5 ± 6.9, and after the program, it was 12.4 ± 6.0. The reduction of 3.2 points was statistically significant (Table 2). The proportion with a high perceived stress level (PSS ≥ 18) before MBSR was 38%. This was reduced to 22% after MBSR (*p* = 0.0032). Among the group with PSS ≥ 18 at baseline, the PSS score was significantly reduced by 6.4 score points from a pre-mean score of 22.6 ± 3.6 (Table 3). For the SCL-5, the pre-score was 2.1 ± 0.6 in the total study population (Table 2) and 2.5 ± 0.6 in the subgroup with PSS ≥ 18 at baseline (Table 3), which were also statistically significantly reduced after MBSR to be 1.8 ± 0.5 and 2.1 ± 0.5, respectively. The scores of the FFMQ also improved statistically significantly by, 6.0 score points in the total study population (Table 2) and by, 7.7 score points in the subgroup with PSS ≥ 18 at baseline (Table 3). The reference MBSR RCT, with FFMQ as an outcome measure, had a between-group effect size of *d =* 0.47 with a within-intervention group effect size of *d* 1.00 (Robins et al. 2012). In comparison, the Cohen’s *d* in our study was 0.67 for the total study population and 1.09 for the subgroup with PSS ≥ 18 at baseline (Table 4). The reference RCT that had used PSS as an outcome measure had a between-group effect of *d* = 0.64 and a within-intervention group effect of *d =* 1.00 (Nyklicek and Kuijpers 2008). In our study, the Cohen’s *d* was 0.50 for the total study population and 1.47 for the subgroup with PSS ≥ 18 at baseline (Table 4). Finally, the reference RCT that had used SCL-5 as an outcome had a between-group effect of *d =* 0.57 and a within-intervention group effect of *d* = 0.77 (de Vibe and Moum 2006). In our study, the Cohen’s *d* was 0.48 for the total study population and 0.81 for the subgroup with PSS ≥ 18 at baseline (Table 4).

**Table 1:** Characteristics of 132 self-paid participants in MBSR in Denmark, 2016

Gender, female (%)^a^	95 (79)
Age, mean (SD)^a^	45 (10.4)
Education (%)^a^	
≤ 11 years	3 (3)
> 11 < 16 years	27 (22)
≥ 16 years	91 (75)
Living alone (%)^a^	23 (19)
Living with a partner (%)^a^	81 (66)
Living with children/adolescents (%)^a^	39 (32)
Living with children/adolescents, no partner (%)^a^	25 (21)
Employment status^a^	
Employed (%)	92 (76)
Unemployed (%)	8 (7)
Student (%)	1 (1)
Retired (%)	1 (0.8)
Disability pensioner (%)	2 (2)
Sick leave (%)^b^	14 (14)
Disease, present/earlier (%)^c^	
Asthma	9 (8) / 10 (9)
Diabetes	0 / 0
Hypertension	5 (5) / 7 (6)
Myocardial infarction	0 / 0
Angina pectoris	1 (1) /0 (0)
Stroke	0 / 0
COL	0 (0) / 1 (1)
Osteoarthritis	14 (13) / 0 (0)
Rheumatoid arthritis	1 / (1) / 0 (0)
Osteoporosis	2 (2) / 0 (0)
Prolapse	10 (9) / 8 (7)
Cancer	0 (0) / 5 (5)
Migraine	14 (13) / 18 (16)
Mental disorder ≤ 6 months	2 (2) / 17 (15)
Mental disorder > 6 months	8 (7) / 10 (9)

Missing data ^a^< 10%, ^b^< 26%, ^c^< 17%,

**Table 2:** Changes in perceived stress, symptoms of anxiety and depression and mindfulness after participating in a MBSR program among 132 self-paid participants, DK 2016

Outcome^a^	Pre	Post	Difference (95% CI)	% of change
PSS, mean (95% CI)	15.5 (14.3 to 16.9)	12.4 (11.3 to 13.4)	−3.2 (−4.2 to −2.1)^b^	21
PSS ≥ 18, % (95% CI)	38 (31 to 45)	22 (15 to 29)	−16%-points (−26 to −6)^c^	
SCL-5, mean (95% CI)	2.1 (1.9 to 2.2)	1.8 (1.7 to 1.9)	−0.3 (−0.4 to −0.2)	14
FFMQ-15, total, mean (95%CI)	49.6 (47.9 to 51.4)	55.6 (54.7 to 57.1)	6.0 (4.7 to 7.3)^b^	12
Observing, mean (95% CI)	9.8 (9.3 to 10.13	11.0 (10.7 to 11.4)	1.3 (0.9 to 1.6)^b^	13
Describing, mean (95% CI)	11.6 (11.1 to 12.0)	12.2 (11.8 to 12.5)	0.6 (0.3 to 0.9)^d^	5
Acting with awareness, mean (95% CI)	9.3 (8.9 to 9.8)	10.3 (9.9 to 10.6)	0.9 (0.5 to 1.3)^b^	10
Non-judging of inner experience, mean (95% CI)	10.7 (10.2 to 11.3)	12.3 (11.8 to 12.8)	1.5 (1.1 to 2.0)^b^	14
Non-reactivity to inner experience, mean (95% CI)	8.5 (8.0 to 8.9)	10.1 (9.7 to 10.5)	1.6 (1.2 to 2.1)^b^	19

Missing data ^a^< 11%, ^b^ *P* < 0.00001, ^c^ *P* < 0.005, ^d^ *P* < 0.0005

**Table 3:** Changes in perceived stress, symptoms of anxiety and depression and mindfulness after participating in a MBSR program among 50 self-paid participants with Perceived Stress Scale (PSS) score ≥ 18 at baseline, DK 2016

Outcome^a^	Pre	Post	Difference (95% CI)	% of change
PSS, mean (95% CI)	22.6 (21.6 to 23.7)	16.3 (14.8 to 17.7)	−6.4 (−8.1 to −4.6)^b^	28
SCL-5, mean (95% CI)	2.5 (2.4 to 2.7)	2.1 (1.9 to 2.2)	−0.5 (−0.6 to −0.3)^b^	20
FFMQ-15, total, mean (95%CI)	44.1 (42.0 to 46.2)	51.8 (49.7 to 53.9)	7.7 (5.3 to 10.0)^b^	17
Observing, mean (95% CI)	9.0 (8.2 to 9.8)	10.7 (10.0 to 11.3)	1.7 (1.1 to 2.2)^b^	19
Describing, mean (95% CI)	10.4 (9.7 to 11.1)	11.2 (10.6 to 11.8)	0.8 (0.2 to 1.4)^c^	8
Acting with awareness, mean (95% CI)	8.6 (8.1 to 9.1)	9.7 (9.2 to 10.3)	1.1 (0.4 to 1.7)^d^	13
Non-judging of inner experience, mean (95% CI)	9.1 (8.3 to 9.9)	11.1 (10,3 to 12.0)	2.1 (1.3 to 2.8)^b^	23
Non-reactivity to inner experience, mean (95% CI)	7.2 (6.6 to 7.7)	9.3 (8.7 to 9.9)	2.1 (1.4 to 2.8)^b^	29

Missing data ^a^< 10%, ^b^ P < 0.00001, ^c^ *P* < 0.02, ^d^ *P* < 0.002

**Table 4:** Comparison of changes in our study population (*n* = 132) with changes in intervention groups that had been found statistically significant different from changes in control groups in randomized controlled trials evaluating MBSR

Outcome measure	Our study		RCT (*n* = 56)^a^, people interested in learning mindfulness as a means of reducing stress, mean age 46, 51% had a graduate degree, 84% women, USA, 2011. Intervention group (*n* = 20)	RCT (*n* = 57)^b^, people with symptoms of distress, mean age 46, 51% with high education level, 67% women, the Netherlands, 2008. Intervention group (*n* = 29)	RCT (*n* = 144)^c^, people with stress and chronic illnesses, mean age 47, 68% with academic education, 88% women, Norway, 2006. Intervention group (*n* = 102)
	Total (*n* = 132)	Subgroup (PSS ≥ 18) (*n* = 50)			
FFMQ			*d* compared to randomized control group: 0.47, *p* 0.001		
Pre	3.31^d^	2.94^d^	3.12^e^		
Post	3.71^d^	3.45^d^	3.55^e^		
*d* within-group	0.67	1.09	1.00		
PSS				*d* compared to randomized control group 0.64, *p* 0.02	
Pre	1,55^f^	2.26^f^		2.32^g^	
Post	1,24^f^	1.63^f^		1.81^g^	
*d* within-group	0.50	1.47		1.00	
SCL-5					*d* compared to randomized control group 0.57, *p < 0.001*
Pre	2.1	2.5			2.3
Post	1.8	2.1			1.8
*d* within-group	0.48	0.81			0.77

^a^Robins et al. 2012

^b^Nyklicek and Kuijpers 2008

^c^de Vibe and Moum 2006

^d^The average of 15 items

^e^The average of 39 items

^f^The average of 10 items

^g^The average of 14 items

### Post Questionnaire Non-responder Characteristics and Possible Impact on Results

The 69 participants that did not complete the post course questionnaires had higher education levels than the rest of the study population (*p* = 0.012). The majority of post course questionnaire non-responders (43 of 45 who provided data on education level at baseline) reported ≥ 16 years of education. In our study population, 91 of 121 who provided baseline data on education level reported ≥ 16 years of education. There was more missing data on education level for the non-responding group than the responding group (35 vs. 8%). In a sensitivity analysis assuming no change from baseline post MBSR among questionnaire non-responders, the results were as follows: total group, mean change (post minus pre) (95%CI): PSS −2.0 (−2.6 to −1.3), *p* < 0.0000, SCL-5 −0.2 (−0.2 to −0.1), *p* < 0.0000, FFMQ-15 3.8 (2.8 to 4.7), *p* < 0.0000. Subgroup with PSS ≥ 18 at baseline, mean change (post minus pre) (95%CI): PSS −4.1 (−5.4 to −2.8), *p* < 0.0000, SCL-5 −0.3 (−0.4 to −0.2), *p* < 0.0000, FFMQ-15: 4.8 (3.1 to 6.4), *p* < 0.0000.

### Subgroup Analyses

#### Teacher Characteristics

A total of 53 (40%) had participated in a course delivered by one of the two most experienced MBSR teachers (LOF, JP). Comparing this group (1) with the 79 participants (60%) (2) that had participated in the courses delivered by the other eight teachers, the results were as follows: total group, mean difference in pre-post changes (group 2 - group 1) (95%CI); PSS 0.5 (−1.6 to 2.7), *p* = 0.61, SCL-5 0.05 (−0.15 to 0.24), *p* = 0.64, FFMQ-15 2.5 (−0.13 to 5.16), *p* = 0.06. Subgroup with PSS ≥ 18 at baseline, mean difference in pre-post changes (group 2 - group 1) (95%CI): PSS 0.6 (−2.8 to 4.1), *p* = 0.71, SCL-5 −0.01 (−0.39 to 0.37), *p* = 0.97, FFMQ-15 1.6 (−3.2 to 6.3), *p* = 0.52.

#### Gender

Mean changes in women (*n* = 88) minus mean changes in men (*n* = 24) (95%CI) were as follows: PSS 0.8 (−1.7 to 3.3), *p* = 0.51, SCL-5 0.17 (−0.08 to 0.42), *p* = 0.17, FFMQ-15 0.5 (−2.8 to 3.8), *p* = 0.77.

#### Self-reported Mental Disorder

Mean changes in participants with no self-reported mental disorder (*n* = 79) minus mean changes in participants with self-reported disorder (*n* = 28) (95%CI) were as follows: PSS −2.8 (−5.1 to −0.6), *p* = 0.01, SCL-5 0.02 (−0.20 to 0.24), *p* = 0.86, FFMQ-15 −1.2 (−4.3 to 1.9), *p* = 0.45.

#### Attendance

In 6 out of the 15 courses provided in 2016, participant attendance was systematically tracked. Attendance data was therefore available for 49 of the 132 study participants (37%). Among these 49 participants, median attendance was 8 (range 5–8) out of 8 sessions. Only 1 out of the 49 participants (2%) had participated in less than 6 out of the 8 sessions. Forty-two of the 49 with tracked course attendance (86%) had participated in the retreat day. Mean differences in pre-post changes in the groups divided by tracked course attendance (non-tracked minus tracked) were: PSS −0.9 (−3.0 to 1.3), *p* = 0.43, SCL-5 −0.05 (−0.25 to 0.15), *p* = 0.65, FFMQ-15 −0.8 (−3.6 to 2.0), *p* = 0.56.

## Discussion

This study evaluated the effects of attending a self-paid MBSR course in a community setting, delivered by MBSR teachers trained to international standards in Denmark. The results showed statistically significant changes, indicating moderate effect sizes of MBSR on mindfulness, perceived stress, and symptoms of anxiety and depression. For all outcomes, the effects were largest among the subgroup with most perceived stress at baseline. In comparison with within-intervention group effects from reference RCTs that had shown moderate between-group effects, the effects in our total study population were somewhat smaller. This is often seen when translating effects from RCTs to “the real world” with for example more heterogeneous study populations (e.g., different level of perceived stress) as well as more heterogeneous intervention providers (e.g., different level of experience teaching MBSR). Moreover, at baseline the participants in our study had reported a higher level of mindfulness and less perceived stress than the comparison groups from the reference RCTs. This may have meant a ceiling effect for potential changes. Among our pre-defined subgroup with PSS ≥ 18 at baseline, which was more similar to the comparison groups, we found superior effects, especially with regard to PSS (Table 4). Hence, our results were comparable those of the reference RCTs.

Furthermore, in a systematic review of studies evaluating MBSR in non-clinical populations, moderate effects in both pre-post analyses (Hedge’s *g* 0.55) and in between-group analyses (Hedge’s *g* 0.53) were found (Khoury et al. 2015). This also indicates that our results are comparable to those in existing MBSR studies. One study evaluating pre-post changes in mindfulness and perceived stress among a self-paid group of MBSR participants (half were referred by their health care practitioner and others were self-referred) reported that perceived stress decreased significantly, self-reported mindfulness, increased significantly (Carmody and Baer 2008). Interestingly, in that study, the mindfulness facets with the greatest improvements were “observing” and “non-reactivity to inner experience,” which corresponds to our results with largest improvements in “observing,” “non-judging of inner experience,” and “non-reactivity to inner experience” (Table 2, Table 3). Another study also recruited study participants from a self-paid, community-based MBSR program and showed statistically significant pre-post improvement in mental health, as measured by the Short-Form Health Survey, Mental Component Score (Greeson et al. 2011).

The participants in our study, community members who self-referred and self-financed participation in MBSR, were mainly women and represented a socioeconomically advantaged group. They reported higher perceived stress and history of mental disorder compared to the general population. The fact that participation in the MBSR program is a prerequisite for attending the MBSR-teacher education may to some extent explain the relatively high level of education among the participants. However, the participant characteristics in our study were similar to the study population characteristics in our reference trials (de Vibe and Moum 2006; Nyklicek and Kuijpers 2008; Robins et al. 2012) and also in the majority of MBSR trials (de Vibe et al. 2012; Khoury et al. 2015). The subgroup analysis with gender indicated no differences in pre-post changes. According to the National Health Survey in Denmark, a high level of stress was associated with female gender, lower educational level, unemployment, and living alone (Prior et al. 2016). Our results suggest that the self-paid MBSR program does not reach low-income groups with arguable the greatest need for stress management and disease prevention. One other Danish study showed MBSR to be a feasible and cost-effective intervention in patients with somatization disorder and functional somatic syndromes (Fjorback et al. 2013a; Fjorback et al. 2013b). This study population was also mainly female, mean age 40 years, living with a partner, but half of this study population had only mandatory school and in some cases an optional 11th year of school (10. grade), and only a third were employed. These studies suggest that it is feasible to deliver MBSR in a population with lower education levels also. The potential for MBSR in the primary health care system, both in preventing and reducing stress conditions, stress-related diseases has not been well-explored. Prior et al. found a dose-response relationship between perceived stress and mortality, suggesting that stress reduction is not only crucial important in people with the highest perceived stress levels, but also highly relevant in people experiencing moderate stress. The subgroup analysis in our study regarding self-reported current or previous mental disorder indicated that the reduction in PSS was statistically significantly larger in the group without self-reported mental disorder: −2.8 points (95% CI −5.0 to −0.6), *p* = 0.01. One possibility is that those currently experiencing (or have previously experienced) mental health difficulties have more difficulty engaging with the practices that are integral to the program. Although we did not track participants’ home practice, this may be an important avenue for future work (Parsons et al. 2017).

The effectiveness of MBSR highly depends on the acceptability of the program, both to the health care providers that provide the access and for the target group with stress-related problems, independent of factors such as gender and educational level. The challenge with mindfulness is that it can only be understood through direct experience. It requires motivation, persistence, and patience to keep practicing and experiencing the effects. However, these prerequisites are no different from physical training. Self-determination Theory (SDT), a psychological theory on motivation, proposes that persistent high-quality motivation of behavior requires *identified personal endorsement* of doing the behavior (Ryan and Deci 2000). Proponents of SDT also suggest that high-quality motivation, also termed autonomous motivation, can be encouraged by mindfulness (Weinstein and Ryan 2011). Being autonomously motivated to practice mindfulness may lead to healthier behavior, and experience of benefit may further motivate practice. In order to enhance the public health impact of MBSR, a key question is how to support autonomous motivation for participating in the program. This applies to groups with lower education levels and among men generally. Our study, which we conceptualize as a stage 4 study, demonstrated that MBSR can be delivered in a community setting by a group of MBSR teachers, and is of benefit to program participant. Our study also describes some of the characteristics of the participants self-selecting to take part in MBSR offered in the community. We suggest a clear need for future stage 1 research on disseminating MBSR as an intervention (e.g., free access, information, etc.) in order to reach low-resource groups, who require stress management.

### Strengths and Limitations

A strength of the current study was that we evaluated MBSR in geographically different settings, delivered by different MBSR teachers with various professional backgrounds and experience teaching MBSR. Two of the MBSR teachers were co-authors of this paper, but the effect of their courses did not differ significantly from the effect of the courses delivered by the other MBSR teachers. The courses were delivered within typical community settings in Denmark. There were no stringent inclusion/exclusion criteria for participation, or randomization and monitoring of adherence among MBSR teachers and participants. The data collection did not exceed usual quality monitoring of the MBSR courses. Another strength of the current study was the use of intervention groups from reference RCTs for comparison. The effect sizes in our study population were compared with effect sizes in other intervention groups from RCTs with populations similar to our study sample. These RCTs reported significant effects of MBSR compared to control conditions. We suggest that this comparison adds to our initial pre-post analysis, where “regression towards the mean” is a core problem. To our knowledge, this study design has only been used in diabetes prevention implementation research (Absetz et al. 2007).

A clear difference between our study and the reference RCTs was in self-payment. Self-pay is seldom used in RCTs and can be an indicator of a high level of treatment buy in or commitment. Participation in RCTs is often also associated with high motivation. Related to this, we did not systematically track participants’ course attendance, which is a clear limitation of our study. However, the available data showed a high attendance rate, and the course effectiveness did not differ between the groups divided by tracked course attendance. The effectiveness analysis in our study is based on all participants that referred themselves, self-paid a MBSR course delivered by Danish Centre for Mindfulness in 2016 and completed pre- and post measures. We suggest that these results are generalizable to “the real world” and indicate the effectiveness of MBSR in a community setting.

The finding of a higher educational level among the non-responding participants in comparison with the study population should be interpreted with caution, due to the high amount of missing data in the data on education among the non-responding group. Assuming that the non-responding group did not benefit from MBSR participation, the effects for all outcomes remained statistically significant.

This study demonstrated the positive effects of implementing MBSR as a self-referred and self-paid course in a Danish community setting delivered by a group of MBSR teachers. The effects were largest among the participants reporting highest stress at baseline, and the results were comparable with reference RCTs. The participants were mainly women who were middle-aged, with high educational levels, and more perceived stress and a greater history of mental disorder than the general population. Reaching vulnerable, low-resource groups with a clear need for stress management will, however, require other implementation strategies.
